# Future of laser pediatric dentistry: Emerging technologies and clinical applications

**DOI:** 10.6026/973206300212304

**Published:** 2025-08-31

**Authors:** Prachi Kumari, Kanika Singh Dhull, Indira Mysore Devraj, Pankaj Kumar Agrawal, Uttara Anil Thakre, Hasina Khan, Parthkumar K. Thakkar, Pratik Surana

**Affiliations:** 1 Department of Pedodontics and Preventive Dentistry, Kalinga Institute of Dental Sciences, Kalinga Institute of Industrial Technology (KIIT) Deemed to be University, Bhubaneswar, Odisha, India; 2 Department of Pedodontics and Preventive Dentistry, JSS Dental College and Hospital, JSS Academy of Higher Education and Research, Mysuru, Karnataka, India; 3 Department of Oral Pathology and Microbiology, Shri Balaji Institute of Dental Sciences, Mowa, Raipur, Chhattisgarh, India; 4 Department of Pedodontics and Preventive Dentistry, Maitri College of Dentistry and Research Centre, Durg, Chhattisgarh, India; 5 Consultant Pediatric and Preventive Dentist, Hyderabad, Telangana, India; 6 Department of Public Health Dentistry, Karnavati School of Dentistry, Karnavati University, A/907, Adalaj-Uvarsad Rd, Gandhinagar, Gujarat, India

**Keywords:** Caries diagnosis, dental trauma, frenectomy, laser, pediatric dentistry, pulp therapy

## Abstract

Laser dentistry is a promising field within modern, minimally invasive dentistry and it also provides a "child-friendly" approach. This technology has greatly changed pediatric dentistry by offering precise, minimally invasive and comfortable alternatives to traditional methods. Recent advancements, including the integration of artificial intelligence, nanotechnology and innovative delivery systems, have expanded treatment options and improved patient experiences. Thus, this comprehensive review examines the current state, recent innovations and future directions of laser applications in pediatric dentistry, highlighting their impact on care for young patients.

## Background:

The first working laser, the ruby laser, was created by Theodore Maiman in 1960, marking the beginning of laser technology. "Light amplification by stimulated emission of radiation is what the term 'laser' stands for. Light has long been employed as a therapeutic tool and in many medical specialties, laser treatments have been the mainstay of care since the 1970s. However, lasers help regulate both soft and hard tissues in dentistry. The main lasers used for oral surgery include the Neodymium YAG (Nd: YAG) laser, which was created in 1987, as well as argon (Ar) and carbon dioxide (CO2) lasers from the early 1980s [1]. The interaction of laser energy with dental tissues is influenced by wavelength, power settings and tissue properties. Laser energy is monochromatic and different wavelengths have varying absorption rates when interacting with dental tissues [2]. Laser light can either be absorbed or transmitted, depending on the makeup of the tissue. Short-wavelength lasers, such as Ar, diode and Nd: YAG, can penetrate water, however, CO2 wavelengths are absorbed by the apatite crystals in bone and teeth. All biological tissues contain water, which absorbs the Er family of lasers. Melanin and other blood components also absorb short-wavelength lasers. All dental lasers can treat soft tissue, but only the Er family is suitable for hard tissue surgery. Er lasers can remove calcified tissue layers without thermal damage due to their short pulse durations [3]. With the absorption of laser light, the target tissue's temperature increases. At 100°C, the tissue's water starts to boil, vaporizing soft tissue or expanding and destroying hard tissue (ablation). When the temperature exceeds 200°C, the tissue dehydrates and burns due to the air that is present (carbonization), with absorption of all wavelengths by carbon. Lasers primarily affect dental structures through photothermal interaction. The guiding principle for physicians employing a range of lasers to treat various dental disorders is to use the least amount of laser energy necessary to achieve the desired treatment results [3]. Therefore, it is of interest to explore the applications and potential of lasers in pediatric dentistry.

## Classification of laser:

Dental lasers can be classified based on different criteria, including their wavelength, application and the type of laser technology used [4, 5-6]. Here are some common classifications are listed in Table 1 (see PDF).

## Applications of lasers:

Broad application of laser applications in dentistry focuses on diagnostic and therapeutic uses.

## Diagnostic applications:

## Laser fluorescence in caries detection (DIAGNOdent and DIAGNOdent Pen Technologies):

By detecting the fluorescence of bacterial byproducts in carious lesions, DIAGNOdent uses laser fluorescence to identify and measure occlusal and smooth surface caries. It employs a 655 nm diode laser and offers a non-invasive method for early caries detection. Lussi *et al.* (2001) demonstrated that DIAGNOdent has high sensitivity in detecting occlusal caries lesions. The device quantifies bacterial activity, providing numerical readings that correspond to the extent of carious lesions [7]. Aljehani *et al.* (2007) evaluated the DIAGNOdent pen for smooth surface caries detection *in vitro*, finding it effective in quantifying carious lesions with high reproducibility [8]. The DIAGNOdent pen is a smaller, portable version with similar diagnostic capabilities to the original device. Cinar *et al.* (2013) noted that while laser fluorescence provides quantifiable measurements useful for monitoring caries progression, results should be interpreted alongside clinical examination for optimal diagnostic accuracy [9].

## Clinical performance and limitations:

Despite its benefits, DIAGNOdent has limitations. False positives may occur due to stains or calculus and sensitivity varies with calibration. The technique is also influenced by the presence of plaque, calculus and staining, which may produce misleading results if not properly accounted for. However, when used appropriately, DIAGNOdent demonstrates high sensitivity and specificity, particularly for occlusal caries detection, making it a valuable adjunct to visual inspection in pediatric dentistry [7, 8].

## Laser Doppler flowmetry for pulp vitality assessment:

Laser Doppler Flowmetry measures pulpal blood flow to determine tooth vitality, which is particularly useful for traumatized teeth. When compared to alternative vitality assessment techniques for injured anterior teeth, Evans *et al.* (1999) discovered that Laser Doppler Flowmetry was more dependable than traditional pulp testing techniques in newly wounded teeth. Laser Doppler Flowmetry examines blood flow directly, giving a more precise measurement of real pulp vitality than traditional studies that evaluate neuronal response [10]. The technique is especially valuable in cases where conventional electric and thermal pulp tests may yield unreliable results due to temporary loss of sensibility following trauma. This non-invasive objective assessment is particularly beneficial in pediatric dentistry, though specialized equipment requirements and technique sensitivity remain challenges [10].

## AI-Enhanced laser diagnostics:

Artificial Intelligence (AI) represents a transformative technology in pediatric dentistry diagnostic capabilities. AI-enhanced laser diagnostics using various laser systems with AI integration can provide improved diagnostic accuracy, reduced false positives and personalized treatment planning. AI fundamentally changes how diagnostic data is gathered, structured and utilized, thereby enhancing the quality of care for children and adolescents. AI enables pediatric dentists to organize extensive records in a structured manner, ensuring rapid access to critical information about a child's dental history. Deep learning models can extract complex patterns from dental images and radiographs, identifying essential features such as lines, edges, corners and larger patterns in a hierarchical manner. This capability is especially promising for enhancing diagnostic precision and consistency in dentistry [11].

## Therapeutic applications:

Hard and soft tissue applications are broadly divided as given in Table 2 (see PDF), [12, 13, 14-15].

## Hard tissue applications:

Hard tissue dental applications of lasers primarily involve the use of specific types of lasers for procedures on tooth enamel, dentin.

## Caries prevention and management (Laser-Induced Modifications of Enamel Surface):

Nd: YAG laser (1064 nm) modifies the enamel surface to increase resistance to acid dissolution and inhibit bacterial attachment. Mukashev (1991) reported on the use of helium-neon laser radiation for combined treatment and prevention of dental caries in children, finding significant benefits in caries reduction. With laser irradiation, the threshold pH for enamel disintegration is lowered from 5.5 to 4.8, increasing the treated enamel's resistance to acid assaults [16]. The significantly mineralized dental enamel's shape and chemical structure can be changed by laser. Frequencies below 450 mJ/cm^2^ cause the Ca/P ratio to rise, the amount of carbonate and protein to decrease and tri- and tetra-calcium phosphate to develop, all of which point to the participation of photo-thermal mechanisms. This structural modification of enamel contributes to increased resistance against cariogenic challenges. According to researchers, laser treatment results in a decrease in both enamel solubility and permeability. After laser irradiation, the hydroxyapatite crystal becomes more compact, increasing enamel resistance, as the apatite crystal shrinks in size due to the loss of water and CO_2_ [17].

## Cavity preparation and restoration (Er, Er: YAG, Cr: YSGG Laser Applications):

With little thermal harm to nearby structures, carious tissue can be precisely removed using Er: YAG (2940 nm) and Er, Cr: YSGG (2780 nm) lasers. Schein *et al.* (2003) evaluated the dentin-resin interaction pattern after cavity preparation using an Er: YAG laser and found beneficial bonding qualities. The Er: YAG laser lowers the risk of infection and bleeding while performing a wide range of dental treatments with little discomfort [18]. Er: YAG laser use significantly reduced discomfort, anesthetic use and the occurrence and intensity of tooth hypersensitivity, according to a 2024 clinical trial comparing it to a standard dental turbine for the treatment of dental cavities in youngsters. The frequency of tooth fractures did not significantly differ between the two groups; nevertheless, it took longer to prepare cavities with the Er: YAG laser [19].

## Bond strength and restoration interface:

Schein *et al.* (2003) observed unique surface patterns after Er: YAG laser preparation that can enhance bonding of restorative materials [18].

## Curing light activated resins:

Schein *et al.* (2003) discovered that the morphological features of acid-etched, radiographic dentin and the collagen network allowed for optimal monomer transport. In order to assess the pattern of interaction between dentin and resin, SEM was also taken into consideration. Following the preparation of the cavity with an ER: YAG laser operating in a noncontact mode at 250 mJ per pulse, 4 Hz, the components utilize were a focused beam and a fine water mist [18].

## Laser bleaching:

Laser bleaching is a cutting-edge dental procedure designed to achieve powerful whitening effects while minimizing side effects. When utilizing the most effective energy source for this treatment, dental professionals should begin by inquiring about the patient's expectations, lifestyle, medical history and dental habits, ensuring a personalized approach. Additionally, capturing an image of the teeth is essential for assessing changes throughout the treatment process. It's important to discuss potential treatment sensitivities and consider combining both in-office and at-home bleaching methods for optimal results. Antioxidants should be included in the first aid kit to address any immediate concerns during the procedure. Safety protocols must be strictly implemented, which includes providing safety eye protection with orange lenses and appropriate protective clothing for the patient. Rubber dams may also be applied as necessary to isolate the area effectively. Attention to safety is paramount in laser bleaching, as the use of eye protection gear is crucial and operators must have special training to handle the equipment safely. Moreover, hydrogen peroxide-a common bleaching agent-should be handled with extreme caution, employing proper isolation techniques to prevent any adverse reactions [19, 20-21].

## Laser analgesia:

Laser analgesia is a non-invasive, non-destructive technique that reduces or suppresses pain using low-energy irradiation. While the exact causes of its effects are not fully understood, several theories exist. These include the photo-acoustic effect within the gate control pathway, direct effects of laser energy on nerves and nociceptors, modifications to the Na+-K+ pump systems and biochemical changes induced by laser energy. Although techniques are available to achieve a laser-induced analgesic effect and methods to calculate energy doses, it does not provide complete anesthesia, as it cannot entirely suppress all sensations. The primary goal of this application is to reduce the activity of nerves and nociceptors, thereby alleviating pain [22].

## Orthodontic tooth movement:

Orthodontic therapy often requires extended treatment periods, typically lasting 18 to 24 months, which can lead to patient discomfort, reduced motivation and increased risks of periodontal disease, root resorption and enamel demineralization. A promising solution to enhance tooth movement is low-level laser therapy (LLLT). This technique uses low-intensity laser light to stimulate bone remodeling, increase cellular activity and accelerate tooth movement during orthodontic treatment. Studies suggest that LLLT can significantly speed up tooth movement, reduce discomfort and improve overall patient outcomes [23].

## Dental traumatology:

Er, Er: YAG, Cr: YAG, diode and CO2 lasers are the most often employed lasers in dental traumatic injuries for both simple and complex fractures.

## Soft tissue applications:

Soft tissue applications of dental lasers involve using lasers to perform various procedures on the gums and other soft tissues in the oral cavity. Various soft tissue laser applications are discussed below:

## Frenectomy:

A frenectomy is a surgical procedure to remove the frenulum's attachment to the underlying bone, indicated for issues like midline diastema, gingival recession, oral hygiene difficulties, or interference with lip movement. Traditional scalpel techniques carry risks such as bleeding and complications, whereas laser surgery offers advantages like excellent hemostasis, reduced injury to surrounding tissue, less scarring and minimized pain and swelling. This results in greater postoperative comfort and may decrease the need for analgesic drugs, making laser frenectomies a more efficient alternative [24].

## Treatment of aphthous ulcers and herpetic lesions:

Laser therapy for recurrent aphthous stomatitis or isolated aphthous ulcers is one of the simplest and most well-liked laser procedures. Lasers may be able to stop the viral action in its early phases and stop the lesions from returning. Lesions have been effectively treated with the Nd: YAG laser operating in a defocused, non-contact configuration with a free-flowing pulse. Speaking and chewing are made difficult by painful aphthous ulcers. Light contact mode can render lesions insensitive at low wattages in less than four minutes. Lasers in focused mode are utilized to eliminate exposed nerve terminals from the surface of this lesion [25].

## Treatment of mucocele:

Mucoceles are mucus-filled cavities found in the oral cavity, lacrimal sac and paranasal sinuses. They result from two primary conditions: mucus extravasation, caused by mechanical trauma that ruptures salivary gland ducts, leading to mucin spilling into soft tissues and mucus retention, which occurs due to obstruction and dilation of ductal walls without mucin spillage. Treatment options include scalpel incision, surgical excision, marsupialization, micro marsupialization, corticosteroid injections, cryosurgery, laser ablation, sclerosing agents and electrocautery. Soft tissue lasers offer benefits such as minimal bleeding and reduced injury to surrounding tissues [26].

## Indirect pulp capping:

With disinfection reaching a depth of 300 m, pulp capping is superior. Due to decreased heat produced in the pulp chamber, local anesthesia is not necessary when using a laser [11].

## Direct pulp capping:

Laser tissues are advantageous for use in direct pulp capping because they may control bleeding and sterilize surfaces. Er, Cr: YSGG laser running at 20 Hz, 1 W, 15% water and 20% air.

## Pulpotomy:

Traditional pulpotomy agents, particularly formocresol, have long been considered the gold standard for treating primary teeth. However, concerns about its cytotoxicity and potential systemic effects have led to a search for alternative methods. Laser Photobiomodulation (LPBM) has emerged as a promising substitute for pulpotomy procedures. A randomized controlled trial comparing LPBM with formocresol in primary molars showed comparable clinical success rates. Remarkably, the LPBM group achieved a significantly higher radiographic success rate of 94.1%, compared to 58.82% for the formocresol group at nine months post-intervention. Systematic reviews support these findings, indicating that laser pulpotomy techniques not only offer similar clinical and radiographic success rates as traditional methods but also provide added benefits like reduced inflammation and enhanced healing [27].

## Laser assisted root canal disinfection:

Effective disinfection of the root canal system is crucial for the success of endodontic therapy. However, conventional irrigation methods, particularly those using sodium hypochlorite (NaOCl), often struggle to eliminate resilient microorganisms like Enterococcus faecalis, especially given the intricate anatomy of primary teeth. In response to these challenges, laser-assisted disinfection techniques, such as Photon-Induced Photoacoustic Streaming (PIPS) utilizing Er:YAG lasers, have shown enhanced efficacy in promoting root canal cleanliness. PIPS works by using laser energy to generate shockwaves within the irrigant, allowing for deeper penetration and disruption of biofilms. Research has demonstrated that PIPS can achieve a bacterial reduction rate of over 90% against E. faecalis. Additionally, diode lasers have been utilized to augment the antimicrobial activity of NaOCl, further improving disinfection outcomes in endodontic procedures [28].

## Treatment of Dilantin hyperplasia, gingival recontouring in orthodontic patients:

Lasers can eliminate the gingival tissue when replacing teeth where cavities extend below it, stopping bleeding during insertion. When a patient's gingival tissue has hypertrophied due to the use of drugs like Dilantin or when poor oral hygiene occurs when the patient is wearing orthodontic equipment, a laser can be used to remove or repair excessive tissue development [29].

## Benefits of laser dentistry for children:

Pediatric dentists can use lasers to treat both soft and hard tissues with minimal discomfort and pain during minimally invasive procedures. They make dental appointments less stressful and help kids develop a positive dental attitude by reducing the need for injections, getting rid of the vibrations and odors associated with traditional dentistry and being well-liked by both parents and kids. Modern instruments like lasers, which can be a successful preventive and therapeutic approach in children's dentistry, should make the initial dental visit less uncomfortable. With laser technology, soft-tissue operations that once required general anesthesia can now be finished swiftly and safely. Working with children requires understanding age-appropriate skills, functioning and development. Pediatric dentistry is a specialty defined by age, which includes all aspects of child development. Due to minimal invasion, children tolerate laser treatment well, leading to patient cooperation and improved satisfaction among parents, dentists and the children themselves [3, 4].

## Advantages:

Using a laser for polymerization significantly reduces the time required for the process, leading to shorter chair-side durations. This reduction in time is particularly beneficial for improving patient satisfaction, especially for anxious children, as it minimizes their exposure to the dental environment. Additionally, lasers are advantageous in situations where maintaining a dry field for an extended period is challenging. The laser beams maintain their efficacy over distances in hard-to-reach areas, allowing for thicker increments to be cured effectively due to their increased penetration depth. Furthermore, laser use enhances stickiness and helps minimize small leaks, contributing to more efficient and effective dental procedures [30].

## Patient acceptance and behavioral outcomes:

Genovese *et al.* (2008) conducted a study on the efficacy of laser therapy in pediatric dentistry, assessing the tolerance and acceptance of treatments among 50 patients aged 6 to 12 years, all without anesthesia. Using an Er,Cr:YSGG laser (2780 nm) and an Er:YAG laser (2940 nm), a total of 100 treatments were performed. Patient experiences were evaluated with the Wong-Baker modified facial image scale. Results showed a 90% success rate for hard tissue procedures and 63% for soft tissue, with an overall acceptance rate of 75%. The study concludes that Erbium lasers are effective in pediatric dental applications [31].

## Contraindications of laser therapy use:

Pacemaker patients should exercise caution when using laser therapy, as should pregnant women near the uterus. Caution is also advised for individuals with epilepsy or a history of arrhythmia and chest discomfort. Laser treatments should not be applied to thyroid glands or to tumor tissues and benign tumors that have a risk of malignancy. Additionally, those with lupus or on light-sensitive medications should avoid laser therapy. Low-level lasers, or cold-soft lasers, have been used globally for many years, with numerous studies highlighting their benefits in reducing pain and inflammation, enhancing healing quality and speed and boosting the immune system [2, 3, 6 and 7].

## Limitation of laser:

Sachelarie *et al.* highlight that while lasers offer numerous advantages in dentistry, there are significant limitations and challenges to consider when integrating this technology into clinical practice. Among the main concerns are the high costs of equipment, the need for specialized training, and restrictions on treatment applicability, which collectively impede the widespread adoption of lasers in dentistry [32].

## Laser safety:

Dental professionals need to be knowledgeable about the safety of lasers, which includes knowing the risks and hazards connected to laser use, as well as the most recent standards of care and a thorough comprehension of safety control systems. The various dangers that may arise during dental clinical practice can be categorized using the following categories: The risks include risks to the eyes, tissue, respiratory system, fire and explosion, electrical shock, combustion and equipment.

## Conclusion:

Laser dentistry is evolving, with expanded applications beyond periodontics and surgery. While the AAPD supports its use in treating young patients, dental practitioners require further training. Caution and sound judgment are essential as researchers weigh the benefits and drawbacks. Lasers enhance pediatric dentistry by aiding soft tissue surgery, restorative procedures and promoting healthy gingiva.
